# Methodological quality of systematic reviews on interventions for children with cerebral palsy: the evidence pyramid paradox

**DOI:** 10.1111/dmcn.14988

**Published:** 2021-07-11

**Authors:** Stefano Negrini, Pierre Côté, Carlotte Kiekens

**Affiliations:** ^1^ Department of Biomedical, Surgical and Dental Sciences University “La Statale” Milan Italy; ^2^ IRCCS Istituto Ortopedico Galeazzi Milan Italy; ^3^ Centre for Disability Prevention and Rehabilitation and Faculty of Health Sciences Ontario Tech University Oshawaz Ontario Canada; ^4^ Spinal Unit Montecatone Rehabilitation Institute Imola, Bologna Italy

## Abstract

This commentary is on the original article by Kolaski et al. on pages 1316–1326 of this issue.

The paper by Kolaski et al.^1^ poses a series of questions related to evidence generation and appraisal that, albeit not new, cannot be ignored any further. These questions involve scientists, journals, and developers of clinical practice guidelines. However, the most critical, potentially devastating impact is on patients for whom research in medicine is undertaken.

Generating trustworthy evidence is the only way forward for medicine. This was made very clear during the COVID‐19 pandemic, which included issues around rehabilitation.^2^, ^3^ However, research papers cannot be taken at face value: they must be appraised. The most naïve approach is to infer quality by using the primordial pyramid of evidence: the better the design, the lesser the risk of bias, the more reliable the results. Consequently, randomized controlled trials and meta‐analyses are at the top. Nevertheless, the revised evidence pyramid includes blurred boundaries between the different layers:^4^ for example, a bad quality randomized controlled trial is worse than a good quality cohort study. We would argue that a bad quality meta‐analysis is even worse. It can spread false results under the misleading appearance of a reliable study.

The bleak picture on the quality of systematic reviews provided by Kolaski et al.^1^ may be partly attributed to a zealous application of the AMSTAR‐2 criteria. Nevertheless, the message is clear: authors of systematic reviews must do better. In the modern ‘publish or perish’ era,^5^ researchers must rapidly produce results beneficial for their career, funders, and/or employers. Systematic reviews could be regarded as less demanding than clinical trials, mainly when they are not well done. Controlling the methodological quality of these and other papers resides on the shoulders of journal editors and reviewers. Reviewers are the most important, yet weakest link of the peer review chain. Reviewers are made up of researchers and clinicians with similar constraints as the authors: working as unrewarded volunteers and seldom explicitly trained on the job. In clinical fields, clinician‐researchers must manage the time pressure of combining patient care with research activities. Therefore, this link of the chain can easily break, and the quality of reviews suffers. Although editors provide review tools to guide peer reviewers (https://www.equator‐network.org/), these cannot compensate for lack of time, thus impairing the system.

An even more devastating issue is the unreliability of clinical practice guidelines that use flawed systematic reviews. In the knowledge translation process, clinical practice guidelines are essential to inform clinicians, health decision‐makers, and patients. Therefore, the methodology used to conduct clinical practice guidelines must be more rigorous than in primary and secondary research papers. Clinical practice guidelines are expensive to produce, are resource‐intensive, and require the involvement of renowned scientists and research teams who must search the evidence and even more importantly, appraise it properly.

Finally, Kolaski et al.’s paper^1^ confirms that adhering to the Cochrane methodology to conduct systematic reviews leads to high‐quality reviews. However, chairing the Cochrane Rehabilitation Field, we see from within that too many authors do not commit to a Cochrane Review due to the effort and time required to respect the high methodological standards that guarantee the final quality. More rigorous literature searches, standardized application of risk of bias tools, and analyses and reporting of results that incorporate quality appraisal may be more time‐consuming and labour intensive, but will improve future systematic reviews and ultimately have a positive impact on general health.

## References

[1] Kolaski K , Logan Romeiser , Goss KD , Butler C . Quality appraisal of systematic reviews of interventions for children with cerebral palsy reveals critically low confidence. Dev Med Child Neurol 2021; 63: 1316–26. 34091900 10.1111/dmcn.14949

[2] Ceravolo MG , de Sire A , Andrenelli E , Negrini F , Negrini S . Systematic rapid “living” review on rehabilitation needs due to COVID‐19: update to March 31st, 2020. Eur J Phys Rehabil Med 2020; 56: 347–53. 32316718 10.23736/S1973-9087.20.06329-7

[3] Ceravolo MG , Arienti C , de Sire A , et al. Rehabilitation and COVID‐19: the Cochrane Rehabilitation 2020 rapid living systematic review. Eur J Phys Rehabil Med 2020; 56: 642–51. 32705860 10.23736/S1973-9087.20.06501-6

[4] Murad MH , Asi N , Alsawas M , Alahdab F . New evidence pyramid. Evid Based Med 2016; 21: 125–7. 27339128 10.1136/ebmed-2016-110401 PMC4975798

[5] Relman AS . Publish or perish–or both. N Engl J Med 1977; 297: 724–5. 895795 10.1056/NEJM197709292971313

